# A Review on Enhancing the Antibacterial Activity of ZnO: Mechanisms and Microscopic Investigation

**DOI:** 10.1186/s11671-020-03418-6

**Published:** 2020-10-01

**Authors:** Buzuayehu Abebe, Enyew Amare Zereffa, Aschalew Tadesse, H. C. Ananda Murthy

**Affiliations:** Department of Applied Chemistry, School of Applied Natural Sciences, Adama Science and Technology University, P.O. Box: 1888, Adama, Ethiopia

**Keywords:** Metal oxide nanocomposites, Dopants, Antibacterial mechanism, Morphological investigation

## Abstract

**Abstract:**

Metal oxide nanomaterials are one of the preferences as antibacterial active materials. Due to its distinctive electronic configuration and suitable properties, ZnO is one of the novel antibacterial active materials. Nowadays, researchers are making a serious effort to improve the antibacterial activities of ZnO by forming a composite with the same/different bandgap semiconductor materials and doping of ions. Applying capping agents such as polymers and plant extract that control the morphology and size of the nanomaterials and optimizing different conditions also enhance the antibacterial activity. Forming a nanocomposite and doping reduces the electron/hole recombination, increases the surface area to volume ratio, and also improves the stability towards dissolution and corrosion. The release of antimicrobial ions, electrostatic interaction, reactive oxygen species (ROS) generations are the crucial antibacterial activity mechanism. This review also presents a detailed discussion of the antibacterial activity improvement of ZnO by forming a composite, doping, and optimizing different conditions. The morphological analysis using scanning electron microscopy, field emission-scanning electron microscopy, field-emission transmission electron microscopy, fluorescence microscopy, and confocal microscopy can confirm the antibacterial activity and also supports for developing a satisfactory mechanism.

**Graphical abstract:**

Graphical abstract showing the metal oxides antibacterial mechanism and the fluorescence and scanning electron microscopic images. 
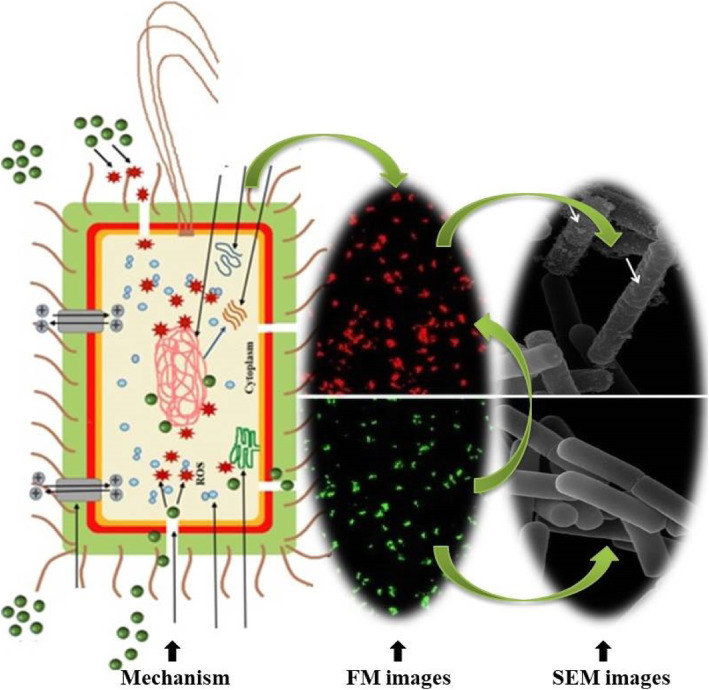

## Introduction

The spread of bacterial infection is a significant offensive threat to human life on this planet. Moreover, the biocompatibility of the synthesized antibiotic has an equivalent rank to assure their safe clinical translations. Recently, nanomedicine has received attention as an antibacterial active material. Metal oxide nanomaterials have been found to exhibit superior antibacterial activity. The antibacterial activity of metal oxide nanoparticles (NPs) is dependent on various parameters such as particle size, surface area, crystallinity, capping/stabilizing agent, morphology, concentration/dosage, pH of the solution, and also the nature of the microorganisms. The smaller the nanoparticles (NPs) and its suitable morphology can penetrate easily through the nanosize pores of the bacteria [1, 2]. Therefore, it is advisable to optimize the parameters as much as possible for the development of novel nanomaterials for the treatment of disease-causing pathogens.

Among many transition metal oxides, ZnO is the most promising inorganic material with a wide range of applications, including (1) as a fillers and rubber compound activator in the rubber industry; (2) as a cream, powders, and dental pastes in the pharmaceutical and cosmetics industry; (3) as UV radiation absorbers in the textile industry; (4) as photoelectronic, field emitters, sensors, UV laser, and solar cell in electrotechnology and electronics industry; (5) as a photocatalyst in photocatalysis. ZnO also has other significant applications such as the production of zinc silicates, for criminal analysis/fingerprint enhancement, and as a packaging material [3]. Compared to TiO_2_, ZnO has an equivalent bandgap energy of 3.3 eV, yet low production cost [4]. It is also known as an II–VI semiconductor based on the positions of Zn and O in the periodic table [5]. The UVA and UVB are characteristics of light energy that has been absorbed by ZnO and generate electrons and holes pairs. It is a versatile inorganic material with a broad range of applications and it was also recorded as a safe material by the U.S. FDA (21CFR182.8991). The generation of ROS and antibacterial activities by iron and manganese oxides were also confirmed [6–10]. Furthermore, iron oxide creates stability against photo/chemical corrosion during the formation of heterojunction with ZnO [11, 12].

The ROS generation, particularly, during characteristic wavelength of light absorption was indicated as the main mechanism of antibacterial activity [13, 14]. The ROS was generated following different mechanisms such as surface adsorption of the bacteria, generation of electron/hole pairs, the reaction of generated pairs with oxygen/water, and formation of different intermediates [15]. However, the release of antimicrobial ions such as Zn^+2^, Mn^+3^, and Fe^+3^ [16] and the electrostatic interaction of NPs with microorganisms [17] was also reported to be the other decent antibacterial mechanism. For the interaction of NPs with the bacteria and generation of ROS, the direct (generation of ROS inside the bacterial cell) and indirect (generation of ROS outside the bacterial cell) methods was reported [18].

Besides its good antibacterial activities of ZnO NPs, many researchers [19–24] have made attempts to improve its ability by forming a heterojunction/composites with metal oxides or by doping of other ions as impurities. Most probably, this improvement is due to the hindrance of the countable ZnO drawbacks such as photo corrosion under UV irradiation [25], electron-hole recombination, lack of visible light absorption, and agglomeration. To understand the antibacterial activities of ZnO-based NPs along with the detailed mechanism, the morphological analysis using microscopic instruments is believed to plays a significant role. The microscopic techniques such as scanning electron microscopy (SEM), field emission scanning electron microscopy (FE-SEM), field-emission transmission electron microscopy (FE-TEM), fluorescence microscopy (FM), and confocal laser scanning microscopy (CLSM) can give detailed information. Therefore, the present review work tries to explore and interpret the antibacterial activity of single, composites, and doped materials with the help of morphological analysis.

### Mechanism of ROS Generation

**Fig. 1:**
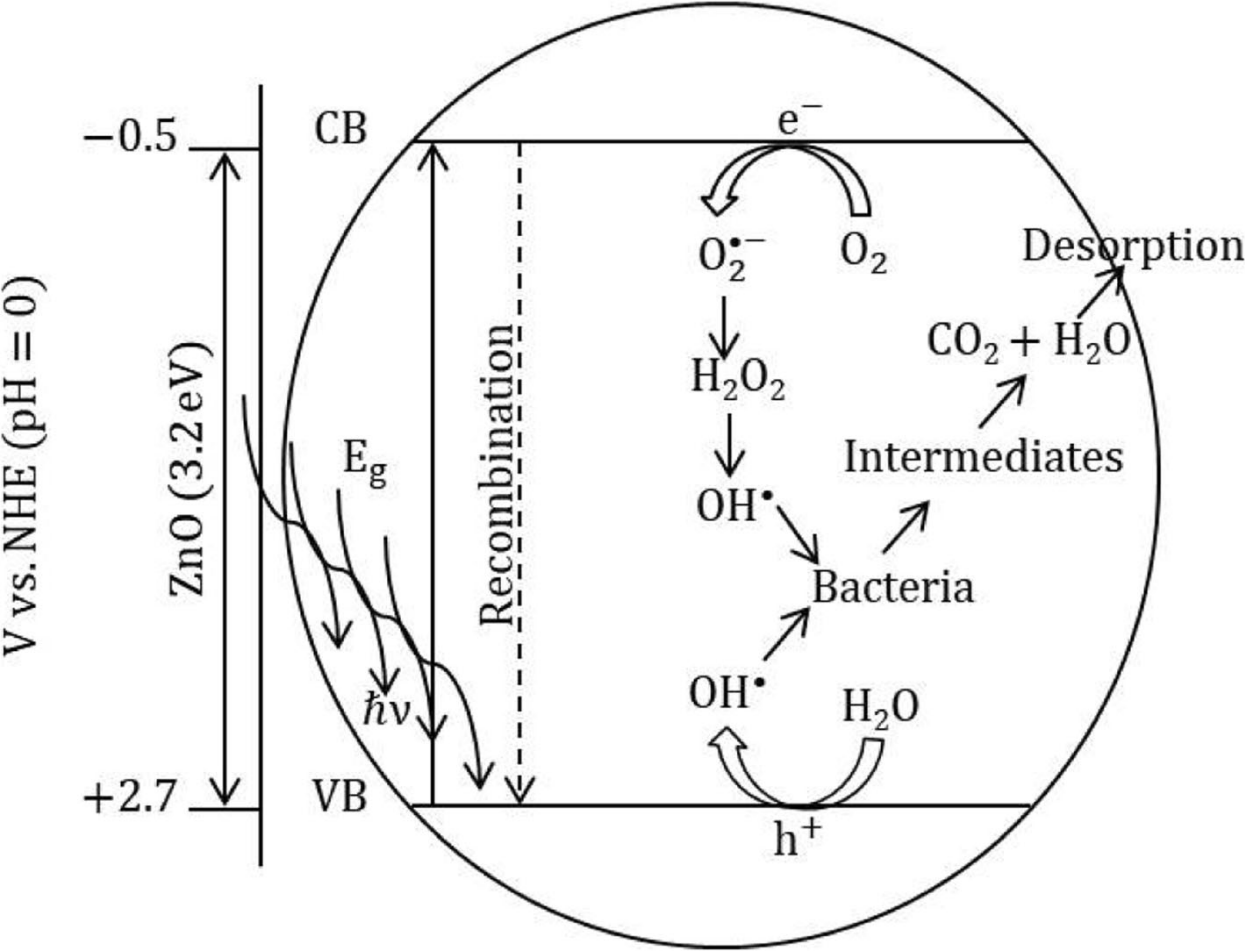
Schematic illustration of the ZnO photocatalytic bacterial degradation mechanism

Metal oxides readily undergo redox reactions catalyzed by light radiation. This is due to their distinctive electronic configuration (such as an occupied conduction band (CB) and a vacant valence band (VB)). Semiconductor metal oxides have a specific bandgap that absorbs the characteristic wavelength of light for the generation of an electron and hole pairs on the CB and VB, respectively. The produced electrons and holes have the probability of recombining in picoseconds or react with other species such as O_2_ and H_2_O adsorbed on the surface of the metal oxides. Through different chain redox reactions, the generated ROS ((hydroxyl radical (OH^•^), hydrogen peroxide (H_2_O_2_), and superoxide (${\mathrm{O}}_2^{\bullet -}$)) believed to degrade the bacterial cell into CO_2_, H_2_O, and other nontoxic minerals [26] (see Fig. 1).

However, this redox reaction is dependent on the VB and CB positions of the metal oxides and redox potentials of the acceptor species. For efficient photocatalysis, the bottom of the CB must be more negative than the redox potential of H^+^/H_2_ (0V compared with NHE), and the top of the VB must be more positive than the redox potential of O_2_/H_2_O (1.23 V compared with NHE) [27, 28]. From the thermodynamic requirement, the oxidation potential of OH^•^, (E^0^(H_2_O/OH^•^) = 2*.*8 V vs NHE) and the reduction potential of ${\mathrm{O}}_2^{\bullet -}$ (E^0^(O_2_/${\mathrm{O}}_2^{\bullet -}$) = − 0*.*28 V vs NHE) should lie with the bandgap of the catalyst. The VB and CB of some metal oxides such as TiO_2_, ZnO, and ZrO_2_ fulfill the requirement and generate the OH^•^ and ${\mathrm{O}}_2^{\bullet -}$ radicals on the surface of the catalyst [29].

### Mechanisms and Antibacterial Activity of Single Metal Oxides

#### Antibacterial Activities of ZnO

The in vitro antibacterial activity of nanomaterials can be performed using different methods such as broth dilution followed by colony count, agar dilution method, disk diffusion assay, microtiter plate-based method, flow cytometry viability assays, and conductometric assay [30]. The antimicrobial activity of ZnO NPs has been tested against both Gram-positive bacteria such as *B. subtilis* and *S. aureus* and Gram-negative bacteria such as *P. aeruginosa*, *C. jejuni*, and *E. coli*. In addition to a thin peptidoglycan layer, Gram-negative bacteria have an outer membrane lipopolysaccharide. This layer acts as a barrier that prevents from entering negatively charged ROS [31]. On the contrary, the cell membrane of Gram-positive has a less negative charge that allows penetration of negatively charged ROS [32]. ZnO shows good antibacterial property on both Gram-positive and Gram-negative bacteria. Yet, the antibacterial activity of ZnO is highly dependent on the particle size. It was reported that decreasing the particle size results in enhancing the antibacterial activity of ZnO [5, 20]. To indicate, Jones et al. compared the antibacterial activities of MgO, TiO_2_, Al_2_O_3_, CuO, CeO_2_, and ZnO against *S. aureus RN6390* bacteria. Among them, ZnO NPs showed significant growth inhibition. To check the effects of ZnO particle size on the antibacterial activities, differently sized ZnO materials, namely, > 1 μm, 8 nm, and 50–70 nm were studied. Compared to the other, the antibacterial activities of small-sized 8 nm ZnO was found to be superior [33].

**Fig. 2:**
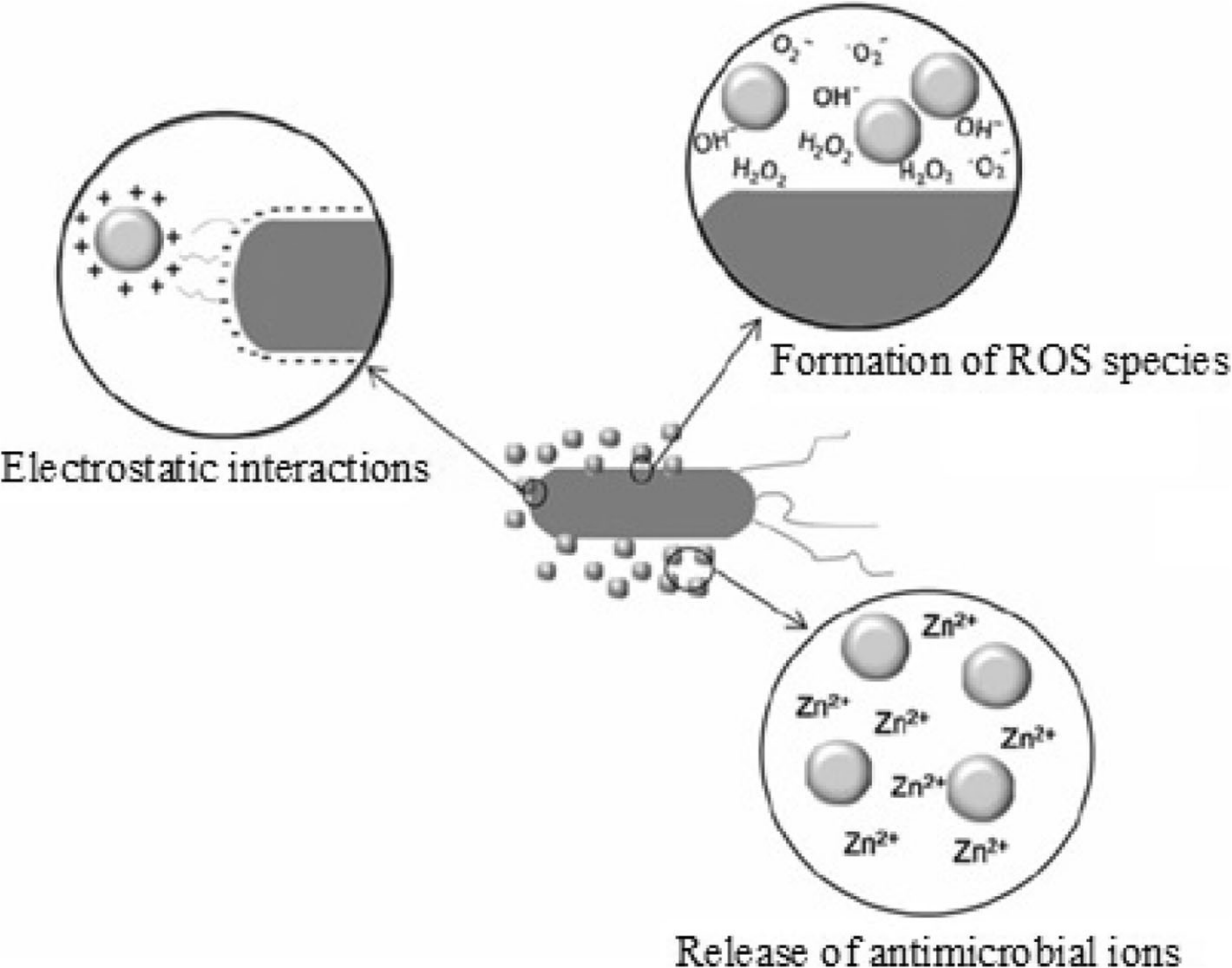
Different mechanisms of antimicrobial activity of ZnO NPs (represented by gray spheres). Reproduced from ref. [30] with permission from Springer Nature

For the antibacterial activities of ZnO, several mechanisms have been proposed. As seen in Fig. 2, the antimicrobial activity mechanism of NPs may follow three mechanisms including the release of antimicrobial ions [30, 34], the interaction of NPs with microorganisms [17], and the formation of ROS by the effect of light radiation [13]. As reported [30], the release antimicrobial ion/solubility of metal oxides is dependent on different factors such as the concentration of metal oxides, time of interaction, and the nature of microorganisms.

### The Release of Antimicrobial ions

**Fig. 3:**
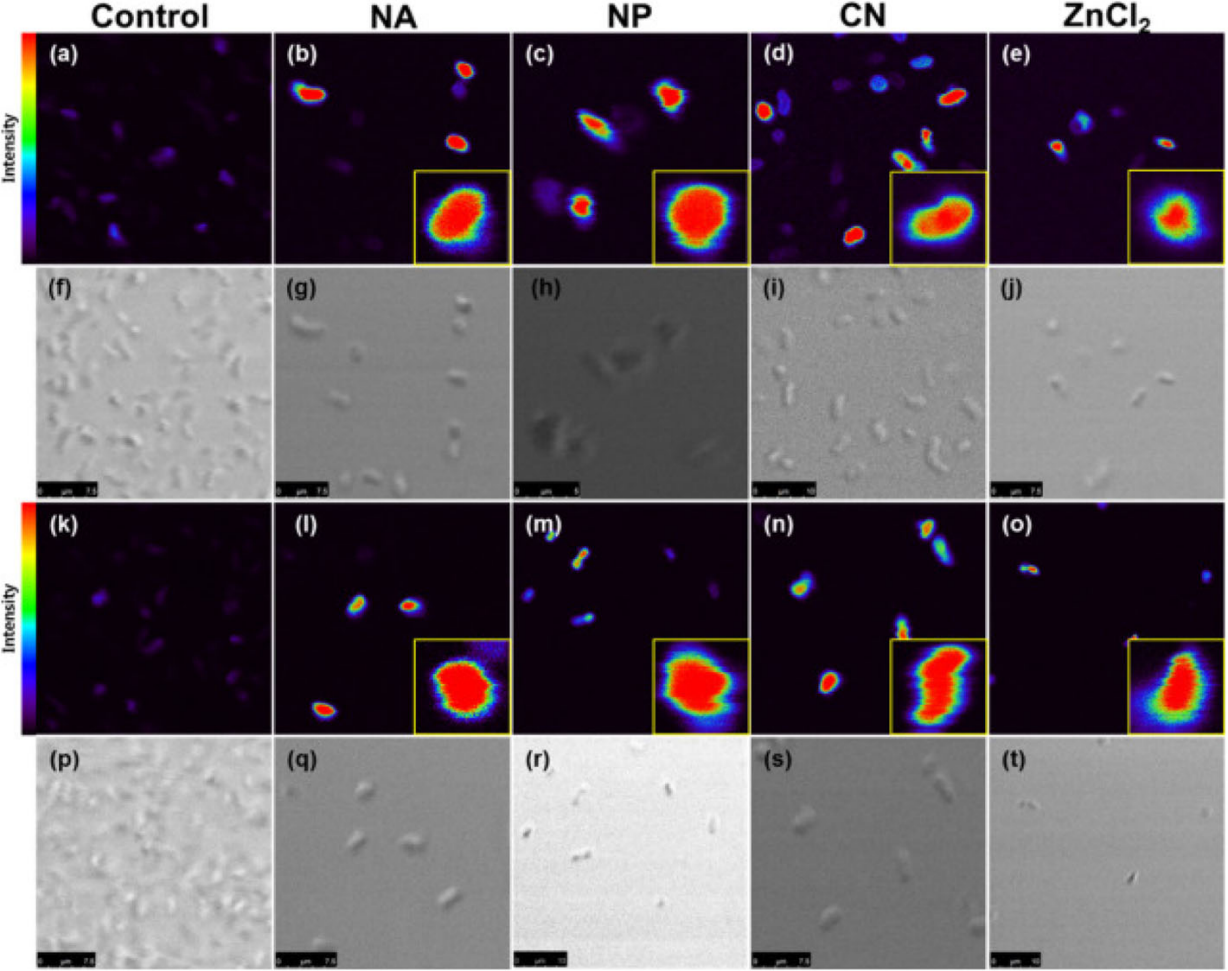
Two-photon fluorescence microscopy and bright-field images of AZn2-labeled (**a**–**j**) *S. aureus* and (**k**–**f**) *K. pneumoniae*. Both species were treated with ZnO NPs (NA, NP, and CN) and ZnCl_2_ of 0.35 mM. Two-photon images were collected at 500–620 nm upon 780 nm excitation with femtosecond pulses. Reproduced from ref. [16] with permission from Elsevier

Joe and his co-workers [16] revealed the release of antimicrobial Zn^2+^ ions under dark conditions. The level of free Zn^2+^ ions was evaluated by Zn^2+^-selective two-photon fluorescent turn-on probe (AZn2) microscopy. Compared to the control (Fig. 3a, k), the approximate mean two-photon excited fluorescence intensities of Zn^+2^ were obtained to be 12 and 6 times higher for *S. aureus* and *K. pneumoniae* bacteria, respectively. The result confirmed the source for Zn^+2^ to be from the dissolution of ZnO. Teichoic acid on the peptidoglycan layer of Gram-positive bacteria and lipoteichoic acid on the outer membrane of the bacteria are the source of negative charges for cell walls. This can also facilitate the attachments of the positively charged ZnO and further dissolution of it.

**Fig. 4:**
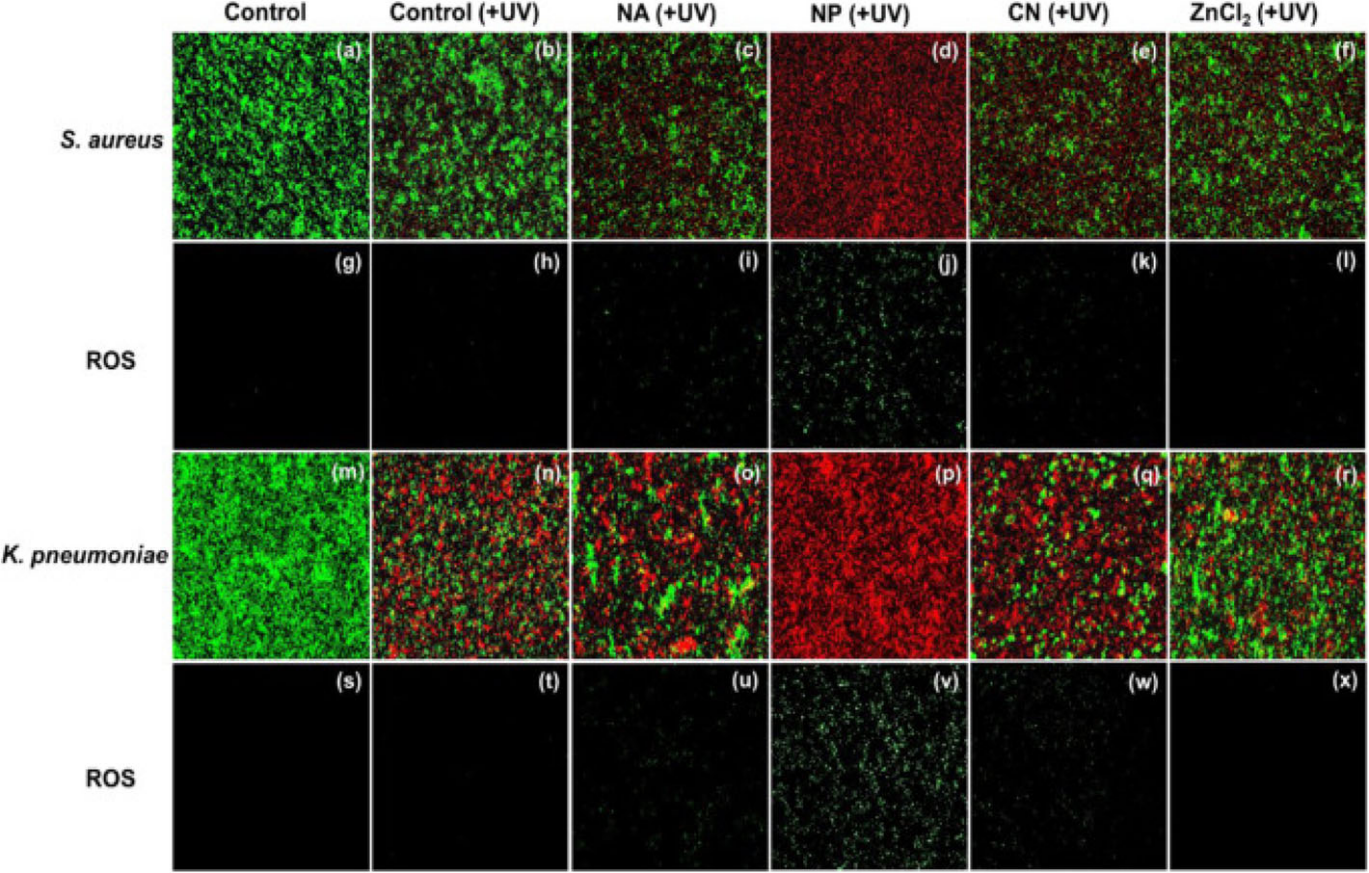
Dual immunofluorescence and ROS staining images for *S. aureus* (**a**–**j**) and *K. pneumoniae* (**k**–**t**) treated with nanoassemblies, NPs, conventional NPs, and ZnCl_2_ (0.35 mM) under dark conditions. Reproduced from ref. [16] with permission from Elsevier

The active and dead cells (calcein-AM (green fluorescence) and PI (red fluorescence), respectively) were confirmed by double immunofluorescence (Fig. 4a–e and k–o). The result also showed the dependency of the size of ZnO and the non-relation of the number of oxygen defect sites on the antibacterial activity. Compared to nanoassemblies and nanoplates, the conventional ZnO NPs showed the highest antibacterial activity on both *S. aureus KCTC No. 3881* and *K. pneumonia KCTC No. 2246* bacteria. The transport of Zn^+2^ ions into the cytoplasmic inner membrane is believed to be through various membrane metalloproteins [35–37]. The release of antimicrobial Zn^2+^ ions in the medium containing microorganisms was also suggested as a reasonable hypothesis about the toxicity of ZnO against *S. cerevisiae* [34].

### The Direct Interaction of NPs with Microorganisms

The direct contact of ZnO NPs with the bacterial cell and production of ROS close to the bacterial membrane that causes damage to bacterial cells has also been suggested to be the other mechanism [17]. First, the cell wall of the bacteria and then the oxidative damage proceeds to the inner cytoplasmic membrane and peptidoglycan layer. Affecting the respiratory activities, slow leakage of RNA and proteins, and rapid leakage of K^+^ ions are believed to be the primary reason for bacterial death. The global negative charge of the bacterial cells at biological pH was occurred due to the dissociation of carboxylic groups [38] and ZnO has positively charged properties at the zeta potential of + 24 mV [17]. The interaction/electrostatic force that occurred between negatively charged bacterial cells and positively charged ZnO lead to disruption of the cell wall and damage occurred by entering into the cell.

The ZnO nanofluid synthesized by Zhang et al. [39] was also reported for direct contact antibacterial activity on *E. coli DH5α* bacteria. It was synthesized by ultrasonication of the commercially obtained agglomerated ZnO powder. Increasing the concentration of ZnO NPs from 0.1 to 0.25 g/l and decreasing its particle size led to an increase in death rate for the bacteria. The result shows considerable damage to the bacterial cell membrane after treatments. This bacterial damage due to the interaction of the bacterial cell and the NPs was further confirmed using electrochemical measurements by a model dioleoyl-phosphatidylcholine monolayer. Polyethylene glycol and polyvinylpyrrolidone were also used as an effective stabilizing agent. As reported, the presence of these capping agents does not have much effect on the antibacterial activity of ZnO nanofluids.

**Fig. 5:**
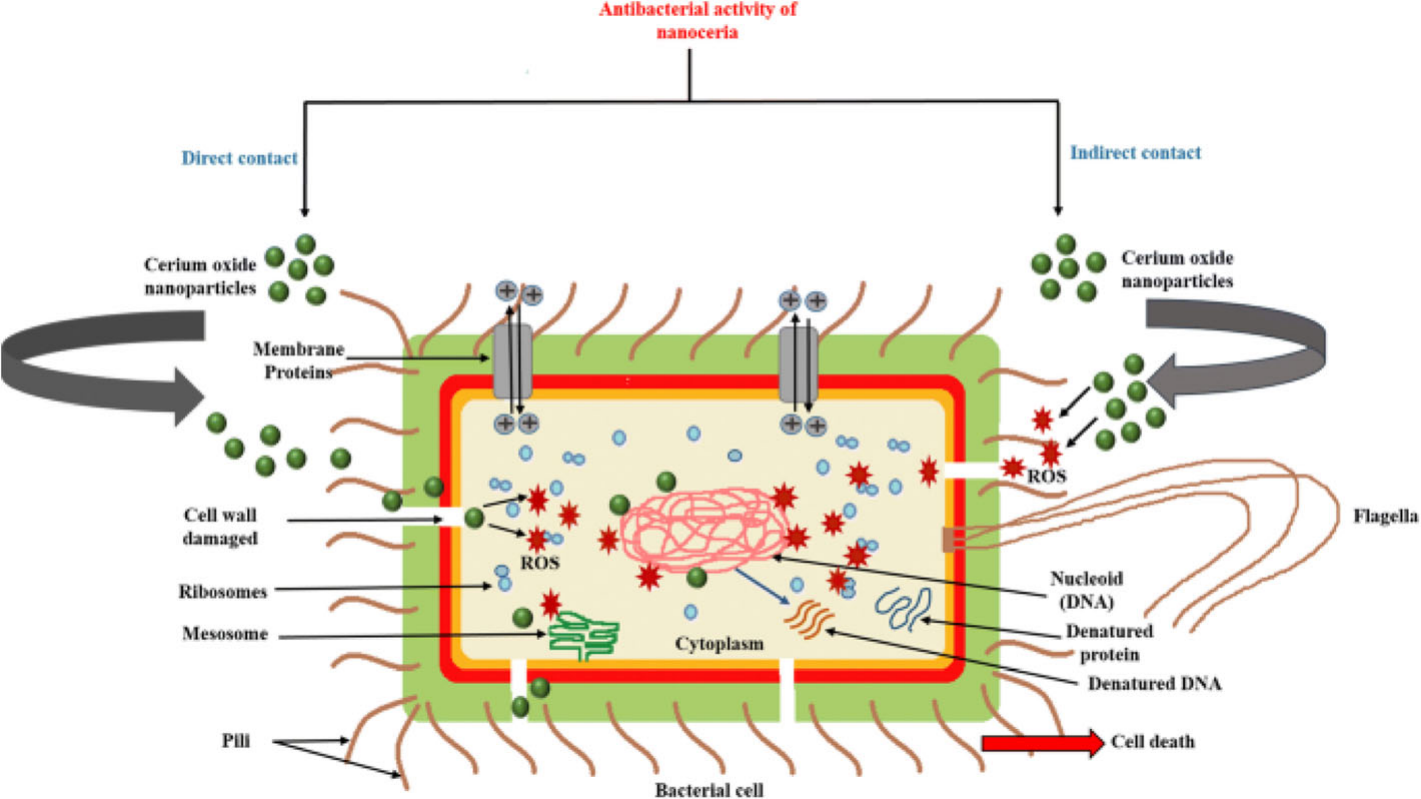
Antibacterial activity mechanism of cerium oxide NPs. **a** Direct contact. **b** Indirect contact. Reproduced from ref. [18] with permission from Springer Nature

Thakur et al. [18] proposed the direct and indirect mechanism for the interaction of cerium oxide NPs with the bacteria cell. As seen in Fig. 5, the direct interaction of cerium oxide NPs led to damage to the cell wall and generates ROS inside. Whereas, the indirect mechanism shows the interaction of cerium oxide NPs with the bacterial environment outside the cell and generates ROS that further enters into the cell by damaging the cell wall. Both mechanisms finally led to cell death by affecting the DNA, ribosomes, and proteins of the bacteria.

### The Formation of ROS by the Effect of Light Radiation

**Fig. 6:**
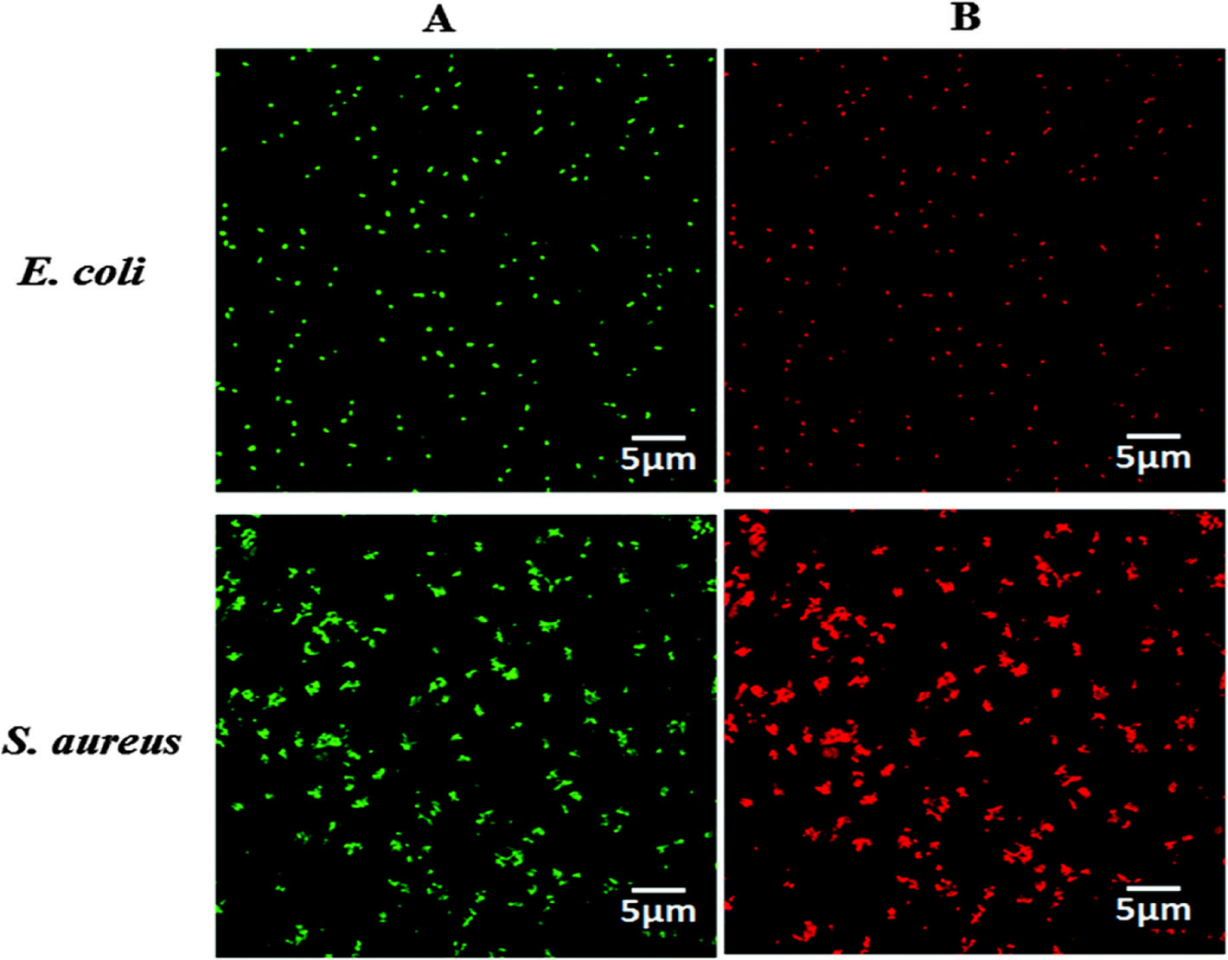
Fluorescence microscopy images of *E. coli* and *S. aureus* treated (**a**) without and (**b**) with ZnO–PVA NPs. Green fluorescence is characteristic of live cells, whereas red fluorescence is due to dead cells. Reproduced from ref. [14] with permission from The Royal Society of Chemistry

On the contrary, this damage due to interaction is believed to arise because of the generation of ROS by the effect of visible/UV light radiation on several studies. Among the other ROS (OH^•^ and ${\mathrm{O}}_2^{\bullet -}$), due to the negatively charged properties of the surface of the bacterial cell, only the H_2_O_2_ reported entering through the cell [13, 32]. The earlier work [14], confirmed the creation of ROS using ESR techniques and their effective antibacterial activity. The novel one-step sonochemical process was used to synthesize the ZnO-PVA composite. The antibacterial activity against *E. coli* and *S. aureus* was conducted using colony-forming units per milliliter method. Compared to ZnO without PVA (~ 80–100 nm), the ZnO NPs synthesized using PVA as a capping agent (~ 4–6 nm) showed enhanced antibacterial activity on both E*. coli* and *S. aureus* bacteria. This confirms the dependency of antibacterial activity on the size of the particles. Using propidium iodide fluorescent dye, the dead cells (red fluorescence) and live cells (green red fluorescence) were identified by confocal laser scanning microscopic images, as seen in Fig. 6. To confirm the ROS generation, the ESR measurements were also carried out using 5,5-Dimethyl-1-pyrroline N-oxide (DMPO) that give DMPO–OH final product after trapping both OH^•^and ${\mathrm{O}}_2^{\bullet -}$ radicals. The ESR spectra show the less amount of DMPO–OH signal produced from the breakdown of the DMPO–OOH adducts which comes from ${\mathrm{O}}_2^{\bullet -}$ radical. Therefore, OH^•^ is reported to be the major contributor to the ROS generation.

Using di-dodecyl dimethylammonium bromide (DDAB) as a surface modifying agent, Viswanathan et al. [40] synthesized greatly dispersed and less aggregated ZnO NPs compared to ZnO NPs synthesized without DDAB. DDAB also improves the affinity of ZnO towards the negatively charged bacteria causing the surface more positive. Compared to pure ZnO, surface-modified ZnO showed better antibacterial activity against both *S. aureus KCCM-11335* and *E. coli DH5α* bacteria. Due to surface charge and dispersion improvement, increasing the amount of DDAB and time of contact, the antibacterial activity also increased. The antibacterial mechanism was proposed to be due to ROS generation. The greater the surface charge, the higher the OH^•^ generation capacity [41]. Compared to single ZnO and cellulose, the enhanced antibacterial activity of ZnO/cellulose nanocomposite was also reported and explained to be due to the smaller crystal size of ZnO in the composite [42]. The agglomerated irregular disc, sheet, and dispersed ZnO on cellulose matrix SEM micrographs were observed for ZnO, cellulose, and ZnO/nanocellulose composite materials, respectively.

However, the mechanism of cell damage by the generation of ROS also becomes contradictory to the study conducted in the dark without light irradiation [43, 44]. As studied by Kadiyala et al. [44], the common ROS generation mechanism of ZnO-NP’s antimicrobial activity was disproved. The fluorescence of 3′-(p-aminophenyl) fluorescein and 2′, 7′-dichlorodihydrofluorescein diacetate was used to quantify the generated ROS. The H_2_O_2_ was used for comparison of ROS generation and antibacterial effectiveness of ZnO-NPs. The result indicated that H_2_O_2_ has greater ROS generation and *S. aureus* killing capability compared to ZnO. This may indicate that for a detailed analysis of the antibacterial activity and suggesting a handy mechanism, applying microscopic techniques that give detailed analysis becomes essential.

### Antibacterial Activities of Fe_2_O_3_ and Mn_2_O_3_

The novel antibacterial activity of Fe_2_O_3_ was also recently verified on different works [6, 7, 45]. Pallela et al. [7] synthesized Fe_2_O_3_ with an average particle size of 16 nm. The morphological study using SEM images showed the presence of spherical nanoclusters. The d-spacing value of 0.27 nm obtained using HRTEM analysis matches with (104) crystal plane of Fe_2_O_3_. The antibacterial activity of Fe_2_O_3_ against *B. subtilis NCIM 2063*, *S. aureus NCIM 2079*, *E. coli 2065*, and *K. pneumonia NCIM 2327* bacteria was tested. Compared to the other, Fe_2_O_3_ shows enhanced antibacterial activity towards *B. subtilis.* However, Fe_2_O_3_ NPs synthesized by Naz et al. confirmed to have less antibacterial activity against *S. aureus*, *P. aeruginosa*, *E. coli*, and *B. subitilis* bacteria [45].

**Fig. 7:**
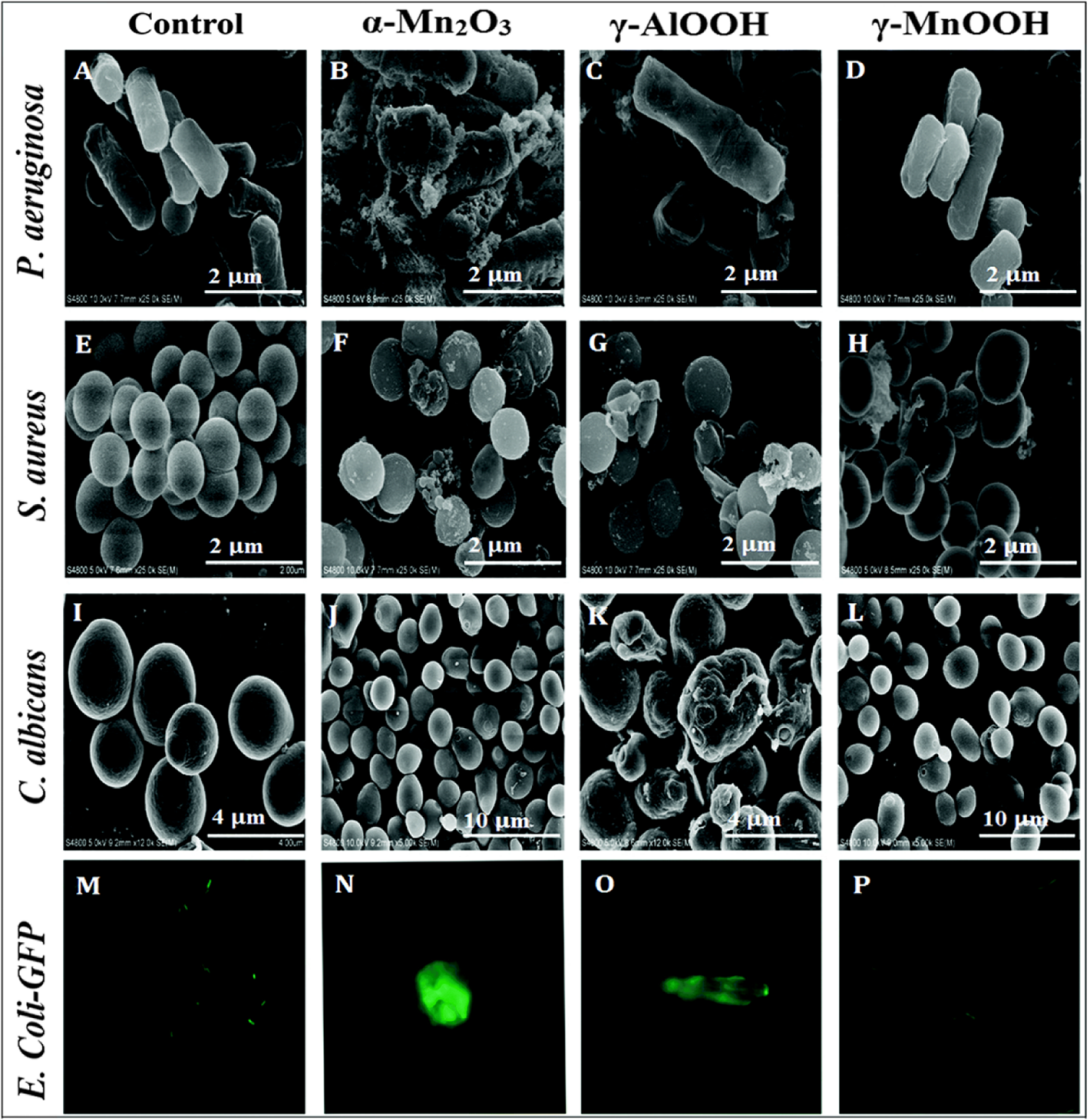
SEM images of untreated and treated bacterial strains using the prepared NRs; where **a**−**d** are for *P. aeruginosa*, **e**−**h** for *S. aureus*, **i**−**l** for *C. Albicans*, and **m**−**p** for *E. coli-GFP* as a control (untreated) and treated with α-Mn_2_O_3_, γ–AlOOH, and γ–MnOOH NRs, respectively. Reproduced from ref. [9] with permission from The Royal Society of Chemistry

In addition to ZnO and Fe_2_O_3_, the novel antibacterial activity of the hydrothermally synthesized α-Mn_2_O_3_ was also reported [9]. Compared to γ-MnOOH and γ-AlOOH, α-Mn_2_O_3_ NRs showed greater antibacterial activity against *S. aureus ATCC23235*, *B. subtilis ATCC23857*, *E. coli ATCC25922*, *B. pertussis ATCC9797*, and *P. aeruginosa Pao1ATCC15692* bacteria. The approximate highest zone of inhibition (ZOI) was determined to be 18 mm on *P. aeruginosa* bacteria. The morphology of untreated and nanorods-treated microbial strains was determined using SEM analysis (Fig. 7). Except for *C. Albicans*, α-Mn_2_O_3_ showed massive deterioration, lethal effect, and morphological changes for all other bacteria. Furthermore, the inhibition of bacterium growth was further confirmed by fluorescence microscopy on *E. coli* (*E. coli*-GFP). The confocal microscopy confirms α-Mn_2_O_3_ to be the highest killing material compared to the other.

The antibacterial activity of chemically and green-synthesized Mn_2_O_3_ was also tested [10]. From SEM analysis, the morphology of chemically-synthesized and green-synthesized Mn_2_O_3_ was determined to be a crystalline cubic structure with 30–50 nm size and spherical with 20–50 nm size, respectively. The antibacterial activity test result showed good bacterial growth inhibition on *E. coli* bacteria compared to *S. aureus*. The size-dependent antibacterial mechanism was reported to be due to the release of positively charged manganese ion and its electrostatic interaction with the negatively charged bacterial cell wall.

### Effects of Different Conditions on the Antibacterial Activity

For enhanced antibacterial activities of metal oxide nanomaterials, optimizing different parameters such as size of the nanomaterials, concentration/dosage, temperature, capping agent, and reducing agent ought to be taken into consideration. To indicate, metal oxides have a large surface area and high surface energy properties. This property facilitates the agglomeration/aggregation to one another and decreases the surface area to volume ratio as well as the antibacterial activity. The aggregation/agglomeration also led to the recombination or quenching of holes and electrons with adjacent aggregate and reduce the generation of ROS [46]. Therefore, researchers are applying a stabilizing agent such as polymers and plant extract [47].

Yamamoto [48] studied the effect of ZnO particle size on the antibacterial activity of *S. aureus* (9779) and *E. coli* (745) bacteria cultured in the Brain Heat Infusion medium. The different particle sizes of ZnO, namely 0.1, 0.2, 0.3, 0.5, and 0.8 *μm* were synthesized by heating the reagent grade ZnO powder at 1400 °C and planetary ball milling process. The antibacterial test of the ZnO powder was conducted by changing the electrical conductivity with bacterial growth. It was found that as the particle size decreases, the antibacterial activity increases. The role of ZnO size on the antibacterial activity of *E. coli W3110* and *S. aureus ATCC 25923* bacteria was also studied [49]. From the SEM morphological analysis, the uniform spherical morphology of ZnO was obtained using polyethylene glycol and the rod-shaped structure using starch as a capping agent. By varying the concentration NaOH, different sizes of the ZnO NPs (40 nm to 1.2 *μ*m) were synthesized. The result indicates the antibacterial activity of ZnO increases as the particle size decrease from micro to the nanorange.

The effects of pH and annealing temperature on the particle size of ZnO during synthesis were effectively studied on Doan et al. study [50]. The ZnO NPs that are used to test the antibacterial activity against *S. aureus* and *E. coli* are synthesized using orange fruit peel extract. The clear spherical-like shape with 10–20 nm-sized ZnO NPs was synthesized at a pH value of 6. The XRD pattern and TEM image analysis result indicate, increasing the annealing temperature results in increasing the intensity of the diffraction peaks and decreasing the crystalline size from 22 nm on 300 °C to 95 nm on 900 °C. This is reported [51] to be due to the reorienting and reducing the number of defects in grain boundaries. Increasing the annealing temperature, results in increasing the bactericidal rate on *S. aureus* bacteria. This has consistent interpretation with the assumption that smaller particle size has superior antibacterial activity. The great effect of the antibacterial activity on the pH value was also observed. Increasing the pH from 4 to 10, results in enhancing the antibacterial activity of ZnO. This is reported to be due to the ability of a generation of more ROS as pH increases. Compared to *S. aureus*, the antibacterial activity against *E. coli* bacteria was obtained to be greater. The great effects of pH on the size and antibacterial activity of ZnO NPs were also reported [52].

The effects of temperature on the size and morphology of ZnO synthesized by pineapple peel extract, and its antibacterial activity was also reported [53]. When the temperature increased from 28 to 60 °C, the size of the ZnO NPs increased from 8–45 nm to the 73–123 nm. FESEM analysis shows a mixture of spherical rod- or flower-rod-shaped structures of ZnO heated at 28 to 60 °C, respectively. Furthermore, as the temperature increases, the agglomeration of the NPs also found to increase. Compared to *Salmonella enterica serovar Choleraesuis* Gram-negative bacteria, ZnO-starch material showed enhanced antibacterial activity on *B. subtilis UPMC1175* Gram-positive bacteria. This is reported to be due to the greater thickness of the cell wall of Gram-negative bacteria that prevents penetration of ZnO into the cell. Decreasing the size of the material by reducing the heating temperature shows improvement in the antibacterial activity. Mohammadi Arvanag et al. [54] also synthesized ZnO/extract (particle size ~ 19 nm) using Silybum marianum L seed and ZnO (particle size ~ 22 nm) NPs using a chemical method for antibacterial activity of *E. coli ATCC 25922*. The experiment was conducted in Muller-Hinton broth media in a concentration range of 0.8–0.05 mg mL^−1^. Both ZnO/extract and ZnO NPs have the potential on preventing the survival of the *E. coli* bacteria as well as completely killing them.

Different concentrations of ZnO NPs incorporated in poly (3-hydroxybutyrate-co-3-hydroxy valerate) (PHBV)/polyethylene oxide (PEO) microfibers were synthesized through electrospinning technique [55]. From the toxicological and biocompatibility point of view of the polymers as active wound dressing materials, the antibacterial activities of PHBV-PEO-ZnO microfibers against *S. aureus NCIM 2654* and *P. aeruginosa NCIM 5032* bacteria were investigated. Compared to control PHBV-PEO, the antibacterial activities of PHBV-PEO-ZnO showed greater ZOI. The tensile strength of the microfiber increases with an increase in the ZnO amount.

The uniqueness of a capping agent towards surface area and antibacterial activity was further confirmed on Gutha et al. [56] study. Compared to single chitosan (CS) and poly(vinyl alcohol) (PVA), the enhanced antibacterial and wound healing properties of chitosan/poly(vinyl alcohol)/zinc oxide (CS/PVA/ZnO) beads was found. The 2*θ* values of separate CS and ZnO, as well as, CS/PVA and CS/PVA/ZnO composites, were precisely confirmed on the XRD pattern. The smooth, nanoflake, and porous morphology for chitosan, ZnO, and CS/PVA/ZnO, respectively, were observed on SEM images. The uniformly distributed ZnO nanorods on the surface of chitosan/poly(vinyl alcohol) were also clearly observed on the TEM image. The highest antibacterial activity on *S. aureus ATCC 29523* bacteria was obtained to be 20 mm on CS/PVA/ZnO material. The effect of various capping agents such as ethylene glycol, gelatin, polyvinyl alcohol, and polyvinylpyrrolidone on the antibacterial activity of ZnO NPs was studied by Akhil et al. [57]. The polydispersed states of ZnO NPs on the capping agent and its hexagonal shape were characterized by FESEM and FETEM. On the contrary, the antibacterial test conducted on *S. aureus MTCC 3160* and *P. aeruginosa MTCC 1688* bacteria show enhanced action for ZnO NPs without a capping agent.

Using citrus lemon extract in a green synthetic approach, Prasad and his Co-workers [58] synthesized 11-nm-sized spherical ZnO NPs. Agar well diffusion assay and broth microdilution assay-MIC and MBC were used for antibacterial activity of ZnO NPs against *K. pneumonia MTCC3384*, *S. aureus MTCC87*, *E.coli MTCC41*, and *P. mirabilis* bacteria*.* As the amount of ZnO NPs dosage/concentration increases, the ZOI also increases. Compared to the other, ZnO NPs showed enhanced antibacterial activity on *P. mirabilis* bacteria.

**Fig. 8:**
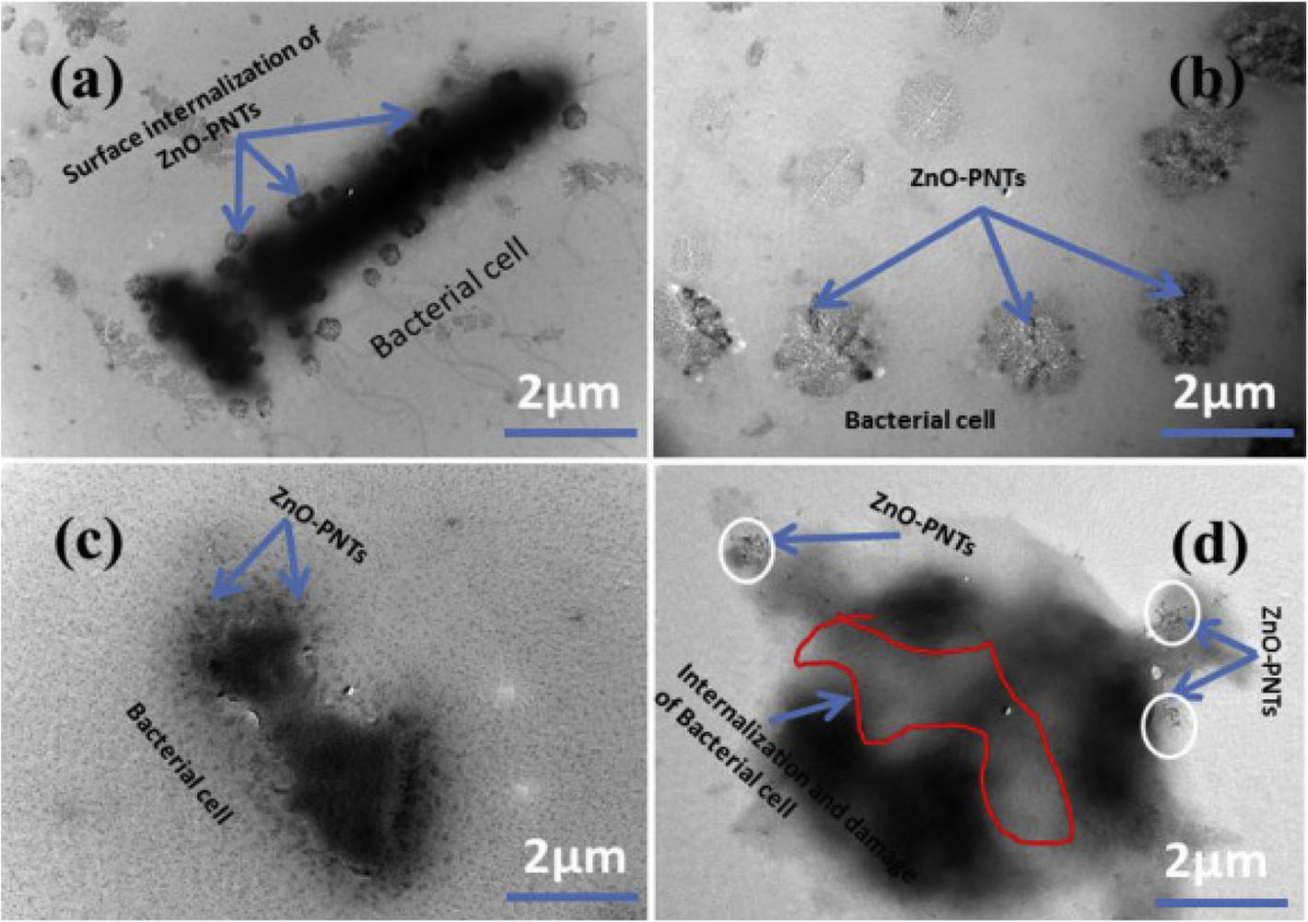
Typical Bio-TEM images of peanut-shaped ZnO with (**a**) *E. coli*, (**b**) *K. pneumoniae*, (**c**) *S.Typhimurium*, (**d**) *S. aureus*. Reproduced from ref. [59] with permission from Elsevier

The direct contact to the surface of cell walls and entering of the ZnO into the bacterial cells (*E. coli*, *K. pneumoniae*, *S. Typhimurium*, and *S. aureus*) by breaking the membrane was shown on the Bio-TEM images analysis (Fig. 8) [59]. The peanut-shaped antibacterial active ZnO nanostructures material was synthesized via a chemical process from a zinc nitrate precursor. Based on their morphological interpretations, cellular cytoplasm leakage and H_2_O_2_ generation are proposed to be the sources for inhibition in the microbial cell membrane. However, bigger particles are not very uniform and have not shown any effective antibacterial action against the tested pathogens. The structure of the nanomaterial was confirmed from the lattice fringes result obtained using HRTEM analysis. The d-spacing value of ∼ 0.526 nm was obtained to be consistent with the wurtzite phase ZnO that develop in the *c*-axis of [0001]. Further crystallinity and consistency with the HRTEM image and XRD pattern were confirmed by selected area electron diffraction (SEAD) pattern. The NPs size and pH of the solution dependency on the antibacterial activity were also indicated. Compared to acidic pH, ZnO NPs synthesized at alkaline pH obtained to have good antibacterial activity.

Using peels of pomegranate (*Punica granatum*), the hexagonal bottom-neck structured of ZnO nanopencils was synthesized for antibacterial study [60]. During the synthesis, different concentrations of Zn(NO_3_)_2_.6H_2_O precursor (2, 3, and 4 g which is coded as ZnO-2, ZnO-3, and ZnO-4, respectively) and 1.5 mL of peel extract were used. The optimum morphology was obtained on 3 g precursor to 1.5 mL peel extract ratio. Increasing the precursor amount to 4 g led to the aggregation of the ZnO NPs. The crystallinity of the material was confirmed by selected area electron diffraction pattern (SAED). The obtained highest ZOI on *S. aureus* bacteria was 21 mm. The synthesized spherical ZnO nanocrystals immobilized onto silicon wafers by self-assembly techniques were informed as antibacterial deactivating materials under dual UV irradiation [61]. Compared to dual UV irradiation without ZnO, the dual UV irradiation in the presence of ZnO NPs showed good *E. coli* bacterial deactivation within 30 s. Furthermore, the spherical ZnO nanocrystals immobilized onto silicon wafers showed enhanced bacterial deactivation within 10 s. The ROS generation was proposed to be the major disinfection mechanism against *E. coli.*

The size-dependent antibacterial activity of ZnO NPs synthesized by taking a different amount of *Mar Ivanios* leaf extract (10, 20, and 50 ml) was also studied by Rufus et al. [62]. As the amount of the leaf extract increases the particle size decreases. The smaller the particle size, the higher the antibacterial activity. The concentration-dependent antibacterial activity of ZnO NPs against *E. coli* and *S. aureus* bacteria shows a good ZOI. As the concentration of the ZnO NPs increase from 25 to 100 μg/μl the antibacterial activity also increases. The synthesized smaller particle-sized ZnO (29 nm) at 100 μg/μl showed 17 and 18 mm ZOI on *E. coli* and *S. aureus* bacteria, respectively. Using solution combustion techniques, Saif et al. [63] also synthesized ZnO NPs using different percentages of *Cordia myxa* leave extract. The disk diffusion antibacterial test of the synthesized ZnO NPs against *S. aureus* and *E. coli* bacteria showed enhanced activity. Brief information on the antibacterial activities of single zinc, iron, and manganese oxides was given in Table 1.

**Table 1:** A set of parameters obtained for antibacterial activities of single zinc, iron, and manganese oxides

Method	Bacteria’s used	Precursors used		Morphological analysis	Highest ZI (mm)	Ref.
		Zn	Other	SEM/TEM/HRTEM/SAED		
Hydrothermal and calcination	*S. aureus* ATCC23235, *B. subtilis* ATCC23857, *E. coli* ATCC25922, *B. pertussis* ATCC9797, *P.s aeruginosa* Pao1ATCC15692	–	KMnO_4_, AlCl_3_.6H_2_O, and sodium dodecyl benzene sulfonates	20 nm mean diameter NRs	18 mm on *P. aeruginosa*	[9]
Green and precipitation	E. coli (ATCC 25922) and S. aureus	–	Mn(AC)_2_·4H_2_O CTMAB and tea extract	Crystalline spherical and cubes NPs with 20–50 nm size	–	[10]
One-step sonochemical	S. aureus and E. coli	Zn(AC)_2_.2H_2_O	–	Dull appearance of PVA polymer with dispersed 4–6 nm ZnO NPs	–	[14]
Soft-solution	S. aureus (KCTC No. 3881) and K. pneumoniae(KCTC No. 2246)	Zn(AC)_2_.2H_2_O	–	ZnO NPs (0001): 60 nm × 10 nm spherical ZnO NAs: 10 nm Conventional NPS: 30 nm	–	[16]
Ultrasonication and milling	E. coli (DH5*α*)	Commercial ZnO	–	ZnO nanofluid		[17]
Nucleation-controlled growth	E. coli and S. aureus	ZnCl_2_	FeCl_2_·4H_2_O	Cubic FCC structure		[32]
–	E. coli (DH5*α*)	Commercial ZnO	Polyethylene Glycol and Polyvinylpyrolidone	ZnO nanofluid		[39]
–	S. aureus KCCM-11335 and E. coli – DH5α	ZnO NPs	Didodecyldimethyl-ammoniumbromide	Flake-like	E. coli > S. aureus and	[40]
In situ solution casting	E. coli and S. aureus	Zn(NO_3_)_2_.6H_2_O	oil palm empty fruit bunches	ZnO dispersed on cellulose matrix		[42]
Green method	*B subtilis (NCIM 2063), S. aureus (NCIM 2079), E. coli (2065), K. pneumonia (NCIM 2327)*	–	Fe(NO_3_)_3_.9H_2_O	16-nm-sized spherical Fe_2_O_3_ with 0.27 nm *d* value with (104) planes	*16 on B. subtilis*	[7]
Green	S. aureus, P. aeruginosa, E. coli and Bacillus subitilis	–	FeCl_3_ and Rhus punjabensis	~ 4-nm-sized spherical NPs	10 on P. aeruginosa	[45]
Green	S. aureus and E. coli	Zn(NO_3_)_2_.6H_2_O	Orange fruit peel	Spherical particles that increases as increasing the annealing temperature	E. coli > S. aureus	[50]
Casting	B. subtilis UPMC1175 and Salmonella enterica serovar Choleraesuis	Zn(NO_3_)_2_.6H_2_O	Starch	Spherical and rod-shaped structure	15 on *B. subtilis*	[53]
Green and chemical method	*E. coli* ATCC (25922)	Zn(NO_3_)_2_.6H_2_O	–	spherical crystalline NPs	–	[54]
Electrospinning	*S. aureus* (NCIM 2654) and P. *aeruginosa* (NCIM 5032)	Zn(AC)_2_.2H_2_O	Poly (3-hydroxybutyrate-co-3-hydroxyvalerate) and polyethylene oxide	2.04–2.2 μm diameter ZnO reinforced microfibers	2.2–3.1 μm rod	[55, 64]
Hydrothermal method	S. aureus (ATCC strain 29523) and E. coli (ATCC strain 29522)	Zn(AC)_2_.2H_2_O	Chitosan and PVA	uniformly distributed ZnO on the surface of the CS/PVA polymer	20 on S. aureus	[56]
Co-precipitation	S. aureus and P. aeruginosa	ZnSO4	Ethylene glycol, gelatin, polyvinyl alcohol, and polyvinylpyrrolidone	ZnO NPs dispersed on capping agent and its hexagonal shape	–	[57]
Green synthetic strategy	*K. pneumoniae (*MTCC3384), *S. aureus (*MTCC87), *E. coli (*MTCC41), *P. mirabilis*	Zn(AC)_2_.2H_2_O	–	11-nm-sized semi-crystalline ZnO with 0.19 nm *d*-spacing value,	18 on *P. mirabilis*	[58]
Chemical process	E. coli, K. pneumoniae, S.Typhimurium, S. aureus	Zn(NO_3_)_2_.6H_2_O	–	∼ 0.526 in the *c*-axis [0001] direction	–	[59]
Pomegranate peels (punica granatum)	E. coli and S. aureus	Zn(NO_3_)_2_.6H_2_O	–	50 nm hexagonal bottom-neck nanopencils	21 on S. aureus	[60]
Green synthesis (Anacardiumoccidentale)	E. coli and S. aureus	–	FeCl_3_	26–60-nm-sized irregular shape	*–*	[62]

*AC* acetate, *S*_*BET*_ specific surface area (m^2^/g), *p*_*V*_ pore volume (cm^3^/g_)_, *P*_*d*_ pore diameter (nm), *d d*-spacing (nm), *p* crystal plane, *ZI(mm)* zone of inhibition in millimeter, *CTMAB* cetyltrimethylammonium bromide

### Antibacterial Activity of Binary and Ternary ZnO-Based Materials

Compared to single metal oxide nanomaterials, forming a binary, ternary, or more heterojunctions can enhance the surface area to volume ratio and diminish the recombination of electron/hole pairs due to the synergistic effect. The heterojunction can be made either with the same or different bandgap metal oxides. It may form either in the n–n or p–n procedure for binary metal oxides and either p–n–n or n–n–n approach for ternary heterojunction [15, 65].

Bhushan et al. [66] synthesized α-Fe_2_O_3_/NiO composites using a co-precipitation method. Compared to pure α-Fe_2_O_3_ and NiO, the enhanced antibacterial activities of α-Fe_2_O_3_/NiO binary nanocomposites were confirmed. Compared to NiO, α-Fe_2_O_3_ shows a greater inhibition zone. The antibacterial activity of the composite increases as the concentration of NiO increases. The results of the disc diffusion assay establish the susceptibility order of the exposed bacteria against Fe/Ni oxide composite NPs to be *E. coli > B. subtilis > S. aureus > S. typhi*. The ZnO-CuO composites materials synthesized by solution combustion techniques using *colotropis gigantea* leaf extract also showed good antibacterial activity against *E. coli* and *S. aureus* bacteria. The synthesized spherical and hexagonal shaped nanomaterials have a particle size of 10–40 nm [67]. Using *Ricinus communis* L. plant seedless fruit extract as a green synthesis procedure, Panchal et al. also synthesized a granular nanoflakes morphology of MgO clusters, irregular morphology of ZnO, and granular nanoflakes shaped MgO/ZnO nanocomposite materials [19]. The antibacterial activity conducted on *E. coli* and *Klebsiella* photogenic bacteria shows enhanced ZOI for the MgO/ZnO nanocomposite compared to single MgO and ZnO. The obtained highest ZOI on *E. coli* bacteria was 28 mm.

Shimada et al. [68] reported a new mechanical rupture-based antibacterial active ZnO/SiO_2_ binary material that was safe regarding toxicity for normal cells. The ZnO/SiO_2_ nanowire was synthesized by bottom-up approaches. Based on the silicon oxides (SiOx)-based surface, the antibacterial activity of SiO_2_ film substrate and ZnO/SiO_2_ nanowire was evaluated against bare glass substrate (Eq. 1). The result showed superior antibacterial activity value for ZnO/SiO_2_ nanowire substrate compared to SiO_2_ film substrate against *E. coli* bacteria. From fluorescence images using propidium iodide test ZnO nanowire showed high cytotoxicity due to zinc ions, while ZnO/SiO_2_ nanowire showed less cytotoxicity. This cell viability on ZnO/SiO_2_ nanowire is reported to be due to zinc ions elution suppression by the SiO_2_ shell layer.

1 $$ R=\left\{\log \left(\frac{B}{A}\right)-\log \left(\frac{C}{A}\right)\right\}=\log \left(\frac{B}{C}\right) $$

*A*, *B*, and *C* are the average counts of colonies formed on the agar medium just after incubation, after culturing on a bare glass substrate for 24 h, and after culturing on SiO_2_ film substrate or ZnO/SiO_2_ nanowire substrate for 24 h, respectively.

The nZnO/TiO_2_ coated on the Ti substrate was synthesized by hydrothermal followed by a low-temperature liquid phase method with different temperate (50, 70, and 90 °C) [69]. From SEM analysis the shape of the composites was obtained to be rhombic prismatic and aligned in nanoarray. Increasing temperature from 50 to 90 °C results in increased particle size from 50 to 90 nm. Compared to *E. coli*, the size-dependent antibacterial activity of the composites is greater for *S. aureus*. Precious Ayanwale and Reyes-López synthesized 26–34-nm-sized ZrO_2_−ZnO NPs for deactivation of *B. subtilis ATCC 19163, S. aureus ATCC 25923*, *S. mutans ATCC 25175*, *E. coli ATCC 25922*, *K. oxytoca 13182*, and *P. aeruginosa ATCC 27853* bacteria [70]. Among different percentages of the composites and single metal oxides, ZnO showed a greater ZOI. The effects of the weight ratio of the metal oxides were also indicated in Gordon et al. [32] study. Different [Zn]/[Fe] weight ratio of 1:9, 3:7, 1:1, 8:2, and 9:1 was prepared for the formation of a mixture of Fe^2+^ and Zn^2+^. From TEM/HRTEM morphology, the FCC structure of Fe_3_O_4_ and ZnFe_2_O_4_ was confirmed to have the same d-spacing value of 0.298 nm. The result indicates the higher the weight ratio of zinc the higher the antibacterial activity. However, increasing the amount of Fe ratio shows decreasing the antibacterial activity; this is reported to be due to the formation of zinc ferrite (ZnFe_2_O_4_) that has no significant antibacterial activity.

In addition to binary metal oxide composites, due to continuous charge transfer synergy, the ternary heterojunction show enhanced antibacterial activity. Anaya-Esparza et al. synthesized TiO_2_-ZnO-MgO nanomaterials by the sol-gel techniques [71]. Using SEM analysis, the materials were confirmed to have less than or equal to 100-nm-sized semi-globular-ovoid shapes. The antibacterial activity of the composites against *E. coli ATCC 8739*, *S. paratyphi ATCC 9150*, *S. aureus ATCC 33862*, and *L. monocytogenes ATCC 15313* bacteria shows good result. Compared to the composite, TiO_2_ showed poor antibacterial activity on all bacteria (only 5–9 mm inhibition zone). The highest ZOI obtained on *S. paratyphi* bacteria is 18 mm. The ROS generation and electrostatic force interaction were suggested to be the probable antibacterial mechanism that led to bacterial death. Compared to single ZnO, the antibacterial bacterial activity of PVA assisted binary ZnO/Fe_2_O_3_ and ZnO/Mn_2_O_3_ [72] and ternary ZnO/Fe_2_O_3_/Mn_2_O_3_ nanocomposite [20] also confirmed on the author’s earlier work. The nanocomposite materials were synthesized using the sol-gel followed by the self-propagation technique.

**Fig. 9:**
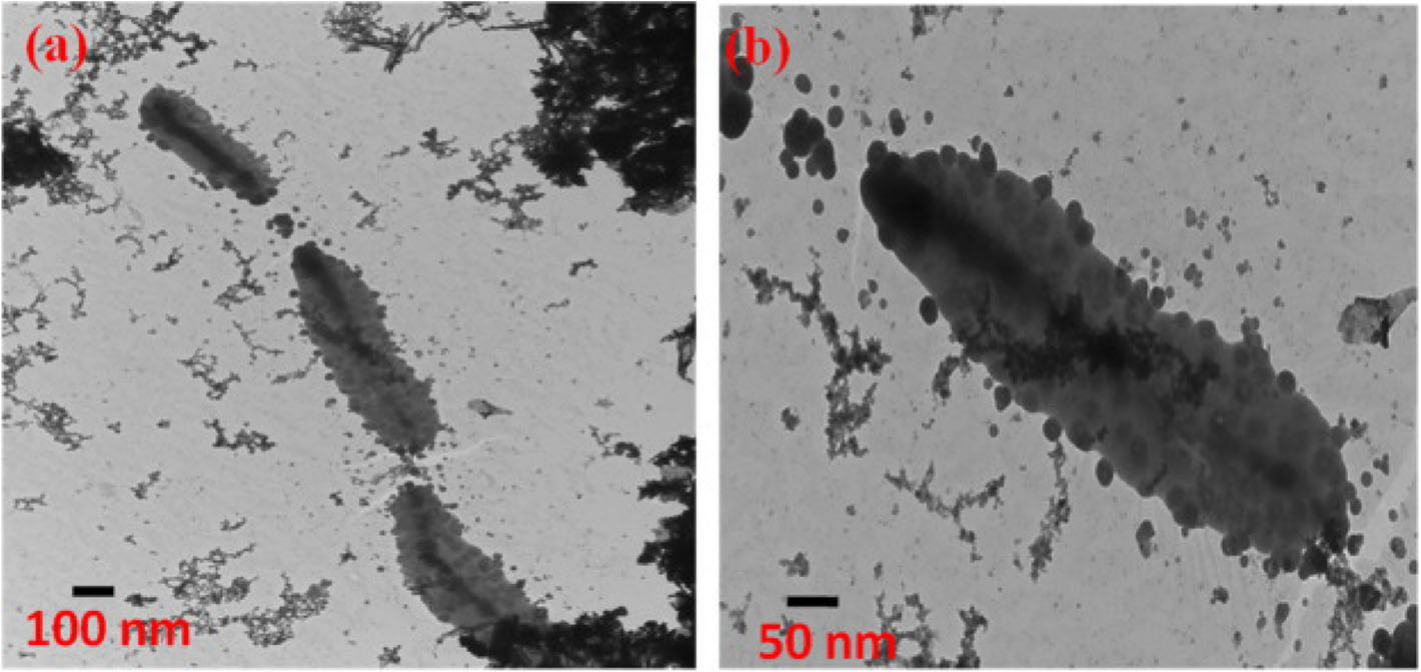
**a** TEM images of *E. coli* mixed with Ag/Fe_2_O_3_/ZnO heterostructure. **b** Ag/Fe_2_O_3_/ZnO heterostructure anchored on the surface of *E. coli*. Reproduced from ref. [73] with permission from Elsevier

Munawar et al. synthesized ZnO-Yb_2_O_3_–Pr_2_O_3_ ternary nanocomposite that showing a highly enhanced antibacterial activity (31 mm) on the *S. aureus* bacteria [74]. The ternary ZnO-Pr_2_O_2_–Yb_2_O_3_ nanocomposite that was synthesized using a co-precipitation technique has porous morphology. The high surface area and porous nature of the material were reported to have good contact with the bacteria. Moreover, the dual-Z-scheme ZnO-Er_2_O_3_-Yb_2_O_3_ material synthesized using co-precipitation techniques was also reported to be highly effective on *S. aureus* bacteria [75]. Kaur et al. synthesized ZnO plates/Fe_2_O_3_ rods/Ag NPs composites for the antibacterial activity of *E. coli* bacteria [73]. The antibacterial activity of the Ag/Fe_2_O_3_/ZnO heterostructures studied at different visible light exposure time (30, 60, and 120 min) presented good results. An increase in the concentrations of Ag/Fe_2_O_3_/ZnO nanocomposite from 0 to 2000 *μ*g/mL, results in decreasing the concentration *E. coli*. The generation of ROS was suggested to be the mechanism of bacterial deactivation. The TEM image analysis was used to realize antibacterial interactions with Ag/Fe_2_O_3_/ZnO nanocomposite (Fig. 9).

Paul et al. synthesized a spherical ternary CuO-NiO-ZnO nanocomposite for antibacterial test against *S. aureus* and *E. coli* bacteria [76]. The obtained result from growth curve analysis and colony-forming unit reduction study showed promising antibacterial activity, specifically an enhanced number of colony counts of bacterial strains reduction on *S. aureus* bacteria. FESEM analysis result also shows the effect of CuO-NiO-ZnO nanocomposites that cause rupturing, cracking, and release of intracellular components.

Owonubi et al. investigated the antibacterial activity of CuO, Fe_2_O_3_ (FeO), ZnO, ZnO/CuO-FeO_*x*_ and ZnO-CuOFeO_*x*_ materials (*x* = 0.1, 0.5 g). The antibacterial activity test was conducted against three different bacterial strains. Among them, the synthesized material was more effective on *S. pneumoniae* bacteria. The order of antibacterial activity test were determined to be CuO > ZnOFeO_0.5_CuO_0.5_ > CuOFeO_0.5_ > ZnOFeO_0.1_CuO_0.1_ > Fe_2_O_3_ > ZnOFeO_0.5_ > ZnO [77]. This indicates that the coupling of more metal oxides enhances the inhibition of the bacteria.

### Antibacterial Activity of ZnO-Based Doped Materials

**Fig. 10:**
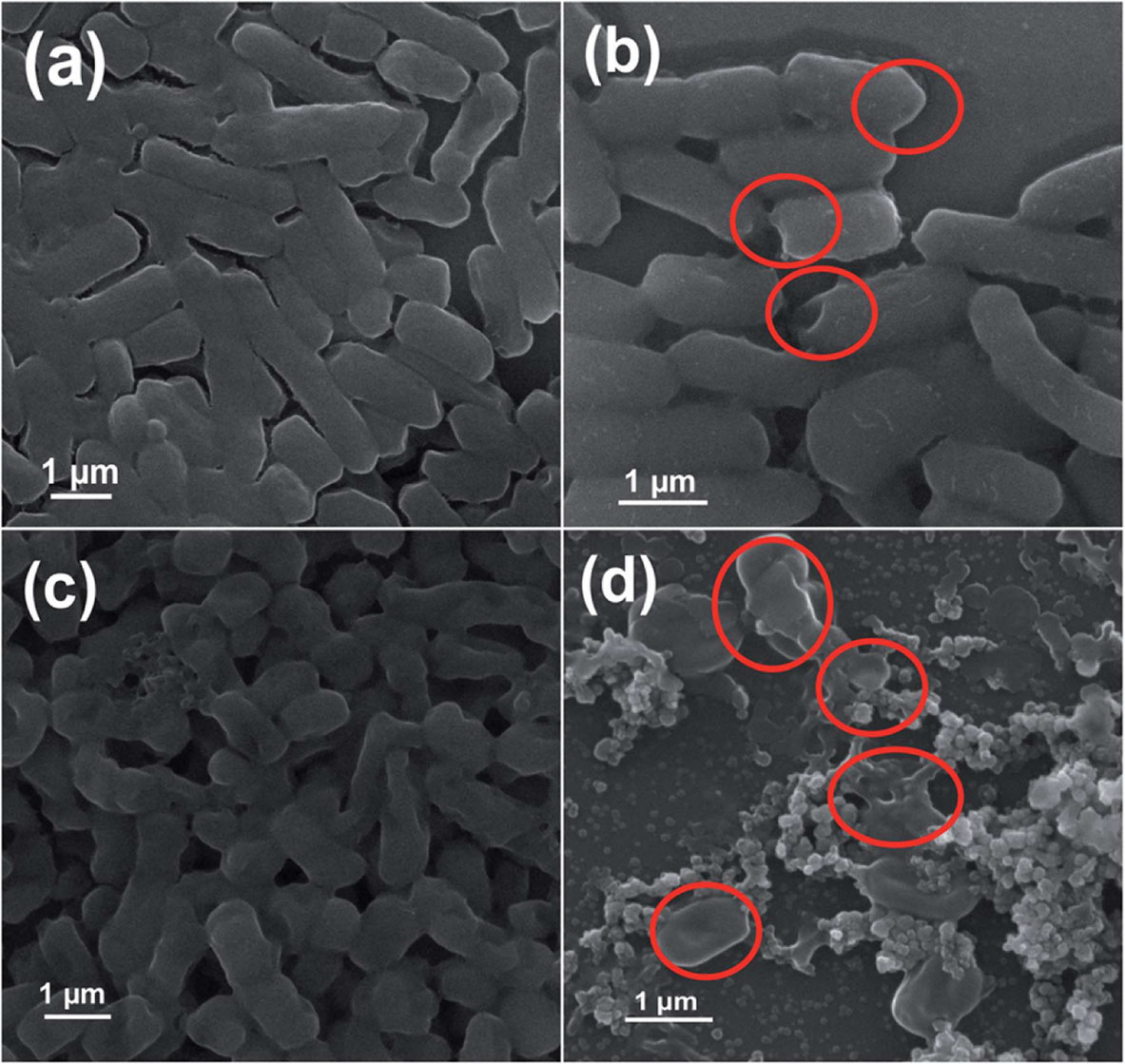
SEM images of bacterial cells. Samples of *E. coli* (**a**) untreated and (**b**) treated with 5NZO. Samples of *A. baumannii* (**c**) untreated and (**d**) treated with 5NZO. Red circles indicate areas of cell membrane disruption. Reproduced from ref. [21] with permission from The Royal Society of Chemistry

Using a one-pot low-temperature solution process, Naskar et al. synthesized Ni^+2^-doped ZnO with 0, 2, and 5 atomic percent (with respect to Zn^2+^) and coded as ZO, 2NZO, and 5NZO, respectively. The antibacterial potential of the synthesized materials was tested against *E. coli ATCC 25922*, *A. baumannii ATCC 19606*, *S. aureus ATCC 25923*, and *Staphylococcus epidermidis* (*S. epidermidis*, *ATCC 12228*) bacteria [21]. The XRD pattern analysis of the materials confirms the successful incorporation of Ni^+2^ into ZnO lattice. The slight 2θ shift towards higher diffraction angle on the XRD pattern was reported to be the good substitution/doping of Zn ion by Ni ions. Compared to ZnO, the highest ZOI was measured on 2NZO and 5NZO Ni^2+^-doped ZnO NPs materials. As seen in Fig. 10, the morphological characterization on *E. coli* and *A. baumannii* bacteria before and after exposure to the 5NZO confirms wrinkling and damage of the bacteria cell (see red circle in Fig. 10).

Using a co-precipitation technique, Thambidurai et al. [78] synthesized NiO-ZnO nanocomposites to evaluate the antibacterial activity on *E. coli*, *S. aureus*, *B. cereus*, and *K. pneumonia* bacteria. The red-shift on UV-vis spectra was also described to be due to the incorporation of Ni^2+^ ions in Zn^2+^ sites of ZnO lattice. The highest antibacterial activity of the composite compared to single ZnO was reported to be due to enhanced surface area and higher ROS generation after modified by Ni^2+^ ions. From the FESEM images, the presence of decorated NPs on the nanorods confirms the formation of NiO-ZnO nanocomposites.

Biogenic like metal-based materials hybridized with active nanomedicine are reported to be safe for normal cells at high concentrations. Compared to free ZnO, due to light-harvesting ability, the as-prepared ZnO-Se showed promising antibacterial activity under light irradiation [79]. The SEM morphology analysis showed the development of ZnO cross bridged Se p- and n-type heterojunction semiconductor composite. Compared to ZnO (3 cm ZOI) on *S. aureus* bacteria, ZnO-Se showed improved results of 5 cm for a long time without re-growing. This long time performance even compared to the standard antibiotic is suggested to be due to the continuous release of ZnO and Se from the ZnO-Se. The performance of ZnO-Se towards the bacteria was further confirmed by live dead cell assay based on propodeum iodide that emits red fluorescence at a certain wavelength.

**Fig. 11:**
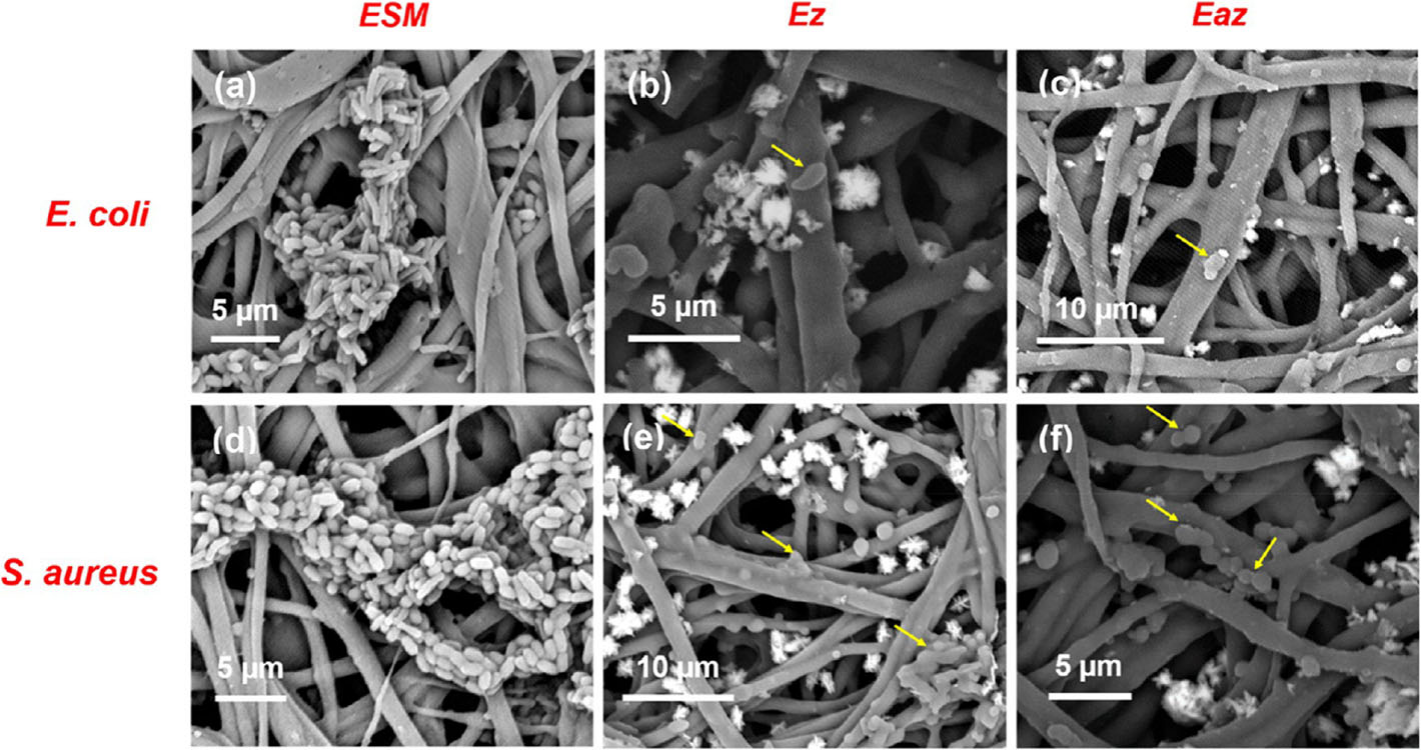
FESEM analysis of the bactericidal activity of nanobiocomposites and bare ESM. **a**, **d** Morphology of *E. coli* and *S. aureus* on bare ESM. **b**, **e** Efficiency of Ez towards *E. coli and S. aureus*; (c and f) efficiency of Eaz composites on the bacteria. Reproduced from ref. [23] with permission from the American Chemical Society

Using a sonochemical approach, Ray et al. synthesized eggshell membrane-loaded microfibrous ZnO/silver NPs matrix that has a good and sustainable release profile for efficient bactericidal activity [23]. After growing the ZnO nanoflowers on the extracted eggshell membrane (ESM), it was further decelerated with Ag NPs. The decoration was conducted by adding a different volume of pre-synthesized colloidal solution of silver NPs (1, 2, 3, and 4 mL). The FESEM samples show the successful embedding of ZnO nanoflowers and further decoration of Ag-NPs on the microfibrous eggshell membrane (ESM). The antibacterial activity of ESM, Ez, 1-Eaz, 2-Eaz, 3-Eaz, and 4-Eaz against *E. coli*, *P. aeruginosa*, *S. aureus*, and *B. subtilis* bacteria was conducted. The 1-Eaz, 2-Eaz, 3-Eaz, and 4-Eaz represents the 1, 2, 3, and 4 mL of silver colloidal solution used to form Ag-loaded ESM/ZnO composites. The obtained highest ZOI on *P. aeruginosa* bacteria is 2.85 cm^2^. The FESEM analysis was conducted to understand the mechanism of antibacterial activities of the nanocomposites (see Fig. 11). Figure 11a, d shows *E. coli* and *S. aureus* bacteria, respectively, migrating along the edges of ESM. The FESEM analysis was also conducted by withdrawing the ESM and composite samples from the plate count experiment. Figure 11c, f shows the distortion of the cell membrane. From ICP−MS analysis, the release of Ag^+^ and Zn^+2^ was reported to be the mechanism for bacterial death. The reaction of Ag^+^ ions with the thiol group of bacterial, affecting the respiratory system by Ag^+^, and the reaction of Ag NPs with sulfur and phosphorus to form a complex with the backbone of DNA was suggested to be the countable way for bacterial death [23].

PVA nanofibers incorporated Fe-doped ZnO NPs were synthesized using an electrospinning technique [80]. The morphological study of different weight percentages of Fe-doped ZnO (4, 8, and 12 wt%) loaded on the PVA polymer was conducted using SEM and TEM analysis. The loading of Fe-doped ZnO NPs on the surface of PVA was confirmed on SEM images. As loading increases, the diameters of composite increases compared to the control. From TEM images, the optimum homogenous bead free continuous fiber loading distribution was obtained on the 8% of Fe-doped ZnO. Compared to pure PVA (96 nm), the higher surface roughness of the composite (8% of Fe-doped ZnO) (135 nm) was further confirmed by Atomic force microscopy. The antibacterial activity conducted on both *E. coli* and *S. aureus* bacteria increases with increasing the loading percentage. Compared to *E. coli*, the *S. aureus* bacteria showed greater sensitivity with a 19 mm maximum ZOI.

The antibacterial activity improvement by doping of Mn on ZnO was also studied by Khan et al. The ZnO materials synthesized using *Melastoma malabathricum* (*L*.) leaf extract exhibited agglomerated spherical morphology. The antibacterial activity of the materials was tested on *E. coli ATCC 25922*, *P. aeruginosa ATCC 27853*, *B. subtilis ATCC 6633*, and *S. aureus ATCC 25923* bacteria. Compared to ZnO (9 mm), the antibacterial activity of Mn-doped ZnO (15 mm) shows greater antibacterial activity [22]. Compared to single ZnO, the antibacterial activity enhancement for TiO_2_ doped ZnO was also proved to be better [81].

The antibacterial activity and its mechanism of Er-doped ZnO/SiO_2_ composites were studied on Yang et al. [82] work. The antibacterial activity improvement of ZnO/SiO_2_ due to the presence of the Er dopant was confirmed by taking different Er amounts. The antibacterial test was conducted by measuring the growth rate of the bacteria (OD600 vs time) in a liquid medium. SiO_2_ and Er-SiO_2_ have almost not shown antibacterial activity. The great antibacterial activity starts after the hybridization of ZnO materials with Er-SiO_2_, indicating novel antibacterial activities of ZnO compared to SiO_2_. An increase in Er^+3^ concentrations led to an increase in antibacterial activity. The amount of released Zn^+2^ ions was measured using flame atomic absorption spectrometry. Until the saturation point, Er^+3^ and Zn^+2^ adsorption increase to enhance antibacterial activity. After saturation point, increasing Er^+3^ ions led to replacing Zn ion and decrease antibacterial activity. The morphological change between control and Er–ZnO/SiO_2_ applied was observed by SEM analysis. After applying the materials, the smooth rod-shaped *E. coli* and the smooth spherical shaped *S. aureus* cells were found to damage the bacterial cell. The release of Zn^2+^ was reported to be the main mechanism that causes bacterial cell.

The antibacterial activity of different time UV-irradiated ZnO-coated Mg alloy were studied [83]. Compared to without irradiation and not submerging in simulated body fluid that has cracks, the 24 h UV irradiated and 2 weeks submerged materials show a dense and integrate bone-like apatite type materials without cracks. Compared to Mg alloy alone and ZnO without UV irradiation (UV0h-ZnO), the enhanced antibacterial activity for ZnO coated Mg alloys with UV irradiation and SBF immersion (UV24 h–ZnO/2W-SBF) was reported. On the FE-SEM images analysis after 12 h incubation, an obvious deformation and lysed was observed on both *S. aureus* and *E. coli* bacteria. The H_2_O_2_ generated from hydroxyl radicals on the surface of ZnO and release of Zn ions are suggested to be the probable mechanism for disruption of the cell membrane, leakage of cytoplasm, and bacterial cell death. Brief information on the antibacterial activities of ZnO-based composites/doped and different optimization was presented given in Table 2.

**Table 2:** A set of parameters obtained for antibacterial activities of ZnO-based composites/dopant and different optimization

Methods	Bacteria’s	Precursors used		Morphological analysis	Highest ZI (mm)	Ref.
		Zn	Others	SEM/TEM/HRTEM/SAED		
Co-precipitation	E. coli, B. subtilis, S. aureus, S. typhi.	–	FeSO_4_.7H_2_O and (NiNO_3_)_2_.6H_2_O)	Crystalline 0.25 [110] α-Fe_2_O_3_ and 0.207 [200] NiO	25 on *E. coli*	[66]
Green and combustion	E. coli NCIM-5022 and S. aureus NCIM-505	Zn(NO_3_)_2_.6H_2_O	Cu(NO_3_)_2_.3H_2_O Calotropis gigantea	10–40-nm-sized spherical and hexagonal irregular shapes	9 on *E. coli*	[67]
Hydrothermal bottom-up approaches	E. coli DH5*α*	Zn(AC)_2_.2H_2_O	Tris(dimethylamino)silane	71.7-nm-sized ZnO/SiO2 nanowires	*–*	[68]
Hydrothermal and low temperature liquid phase	S. aureus (ATCC 25923) and E. coli (ATCC 25922)	Zn(NO_3_)_2_.6H_2_O	Tetrabutyl titanate and Ti (TA_2_, China)	50–90-nm-sized nanoarray aligned rhombic prismatic shape	*–*	[69]
Sol−gel	B. subtilis, S. aureus S. mutans, E. coli, K. oxytoca, P. aeruginosa	[Zn(NO_3_)_2_	Zr(C_4_H_9_O)_4_	Cluster of amorphous particles	~ 7 on B. subtilis	[70]
Sol-gel	E. coli ATCC 8739 , S. paratyphi ATCC 9150, S. aureus ATCC 33862 , and L. monocytogenes ATCC 15313	Zn(NO_3_)_2_.6H_2_O	Titanium-(IV) butoxide, and magnesium di-tert-butoxide	~ 100-nm-sized semi globular-ovoid shape	18 on S. paratyphi	[71]
Green	E. coli, Klebsiella	Zn(NO_3_)_2_.6H_2_O	Mg(NO_3_)_2_.6H_2_O and Ricinus communis L.	Granular nanoflakes	28 mm on E. coli	[19]
Co-precipitation technique	E. coli and S. aureus	Zn(NO_3_)_2_.6H_2_O	Yb(NO_3_)_3_.6H_2_O and Pr(NO_3_)_3_.6H_2_O	Highly agglomerated porous materials	31 on S. aureus	[74]
Co-precipitation	S. aureus and E. coli	Zn(NO_3_)_2_.6H_2_O	Er(NO_3_)_3_.6H_2_O and Yb(NO_3_)_3_.6H_2_O	Loosely packed porous morphology	*21 on* E. coli	[75]
Precipitation	E. coli	Zn(AC)_2_.2H_2_O	Fe(NO_3_)_3_.9H_2_O	ZnO nanoplates/*α*-Fe_2_O_3_ nanorods/Ag NPs heterostructure	–	[73]
Co-precipitation	E. coli and S. aureus	Zn(NO_3_)_2_·6H_2_O	Cu(NO_3_)_2_·3H_2_O and Ni(NO_3_)_2_·6H_2_O	Agglomerated spherical NPs	–	[76]
One-pot low-temperature solution	E. coli (ATCC 25922), A. baumannii (ATCC 19606), S. aureus (ATCC 25923), S. epidermidis (ATCC 12228),	Zn(NO_3_)_2_.6H_2_O	NiSO_4_.6H_2_O	2.0 to 3.0 *μ*m in length with 150 to 200 nm in diameters nanoroads	24 on E. coli	[21]
Co-precipitation	E. coli, S. aureus, B. cereus, and K. pneumoniae	Zn(NO_3_)_2_.6H_2_O	Ni(NO_3_)_2_.6H_2_O	Nanorods are decorated by NPs	27 on B. cereus	[78]
Two-step solution-based technique	*S. aureus*	Zn(AC)_2_.2H_2_O	Sodium selenite	ZnO cross bridged Se heterojunction composite	5 cm *on S. aureus*	[79]
Sonochemical	E. coli or P. aeruginosa, S. aureus, and B. subtilis	Zn(NO_3_)_2_.6H_2_O	AgNO3 and CTMAB	2-μm-sized ZnO Nanoflowers and Ag-NPs decorated ZnO		[23]
Electrospinning technique	S. aureus and E. coli	Zn(NO_3_)_2_.6H_2_O	FeCl_3_	Optimizing the homogenous loading of Fe-doped ZnO on PVA	19 on S. aureus	[80]
Green	*E. coli* (ATCC 25922), *P. aeruginosa* (ATCC 27853), B. *subtilis* (ATCC 6633), and S. *aureus* (ATCC 25923).	Zn(NO_3_)_2_.6H_2_O	Mn(AC)_2_·4H_2_O *Melastoma malabathricum* (*L*.)	Agglomerated spherical NPs	15 on *B. subtilis*	[22]
sol-gel	E. coli (CCTCC AB 204033) and S. aureus (ATCC 25923)	Zn(NO_3_)_2_.6H_2_O	Er(NO_3_)_3_·5H_2_O and sodium silicate	Porous with an irregular shape	–	[82]
Aqueous microwave	E. coli and S. aureus	Zn(AC)_2_.2H_2_O	Mg alloy	Crack filled morphology after UV-irradiation	–	[83]
Electro-deposition	S. aureus (MRSA252; ATCC)	Zn(NO_3_)_2_.6H_2_O	TiO_2_ nanotubes	∼ 4 μm length and ∼ 50 nm diameter TiO_2_ nanotubes	–	[24]
self-assembly	*E. coli*	Zn(AC)_2_.2H_2_O	Oleylamine	3-μm-sized spherical cluster	–	

*AC* acetate, *S*_*BET*_ specific surface area (m^2^/g), *p*_*V*_ pore volume (cm^3^/g_)_, *P*_*d*_ pore diameter (nm), *d d*-spacing (nm), *p* crystal plane, *ZI(mm)* zone of inhibition in millimeter

## Conclusions

ZnO is a promising inorganic material with a wide range of applications in varieties of sectors. It has suitable electronic configuration and biocompatible properties to act as an antibacterial active material. Improvement such as heterojunction, doping, and optimizing different conditions resulted in enhancing the antibacterial activity. Forming a heterojunction and doping improves the charge transfer property, surface area, and stability of the materials. However, for accurate charge transfer synergy and recombination hindrance, proper heterojunction is the requirement. Also, optimizing different conditions such as particle size, crystallinity, the concentration of capping/stabilizing agent/precursors, morphology of the materials, concentration/dosage, and pH of the solution also has an imperative role. Therefore, selecting suitable material that forms a proper junction with ZnO and optimizing conditions as much as possible should be achieved. In addition to careful antibacterial test practice measurement, approving the dead/live experiment result by microscopic techniques also become logical. Accordingly, the authors recommend these microscopic techniques that assist in developing an accurate antibacterial mechanism and confirm the antibacterial activity must be given more attention in the future.

## Data Availability

Not applicable

## Abbreviations

ROS: Reactive oxygen species
NPs: Nanoparticles
SEM: Scanning electron microscopy
TEM: Transmission electron microscopy
FE-SEM: Field emission scanning electron microscopy
FE-TEM: Field-emission transmission electron microscopy
FM: Fluorescence microscopy;
CLSM: Confocal laser scanning microscopy
VB: Valence band
CB: Conduction band
NHE: Normal hydrogen electrode
P. aeruginosa: Pseudomonas aeruginosa
C. jejuni: Campylobacter jejuni
E. coli: Escherichia coli
S. aureus: Staphylococcus aureus
K. pneumoniae: Klebsiella pneumoniae
B. subtilis: Bacillus subtilis
B. pertussis: Bordetella pertussis
C. Albicans: Candida albicans
P. mirabilis: Proteus mirabilis
S. Typhimurium: Salmonella typhimurium
S. typhi: Salmonella Typhi
S. mutans: Streptococcus mutans
K. oxytoca: Klebsiella oxytoca
A. baumannii: Acinetobacter baumannii
PVA: Poly(vinyl alcohol*)*
ZOI: Zone of inhibition
